# Sulfated zirconium oxide-decorated magnetite KCC-1 as a durable and recyclable adsorbent for the efficient removal of asphaltene from crude oil

**DOI:** 10.1039/d1ra04560a

**Published:** 2021-07-29

**Authors:** Farhad Bohlooli Shaafi, Alireza Motavalizadehkakhky, Rahele Zhiani, Seyed Mohammad Mahdi Nouri, Malihesadat Hosseiny

**Affiliations:** Department of Chemical Engineering, Faculty of Sciences, Neyshabur Branch, Islamic Azad University Neyshabur Iran; Department of Chemistry, Faculty of Sciences, Neyshabur Branch, Islamic Azad University Neyshabur Iran R_zhiani2006@yahoo.com; New Materials Technology and Processing Research Center, Department of Chemistry, Neyshabur Branch, Islamic Azad University Neyshabur Iran; Chemical Engineering Department, Hakim Sabzevari University Sabzevar Iran

## Abstract

Sulfated zirconium oxide (ZrO_2_/SO_4_^2−^) as a highly durable acidic reagent was immobilized on magnetite KCC-1 nanoparticles (Fe_3_O_4_@SiO_2_/KCC-1@ZrO_2_/SO_4_^2−^ NPs), and the resulting hybrid was used as a highly efficient recyclable adsorbent for the adsorption and removal of asphaltene from crude oil. The presence of ZrO_2_/SO_4_^2−^ groups not only promotes the adsorption capacity, but also helps recycle the adsorbents without any significant efficiency loss arising from its high chemical resistance. The results showed an obvious synergistic effect between the magnetic core (Fe_3_O_4_ NPs), fibrous silica (KCC-1) and the sulfated zirconium oxide groups with high correlation for asphaltene adsorption. The effective parameters in asphaltene adsorption, including initial asphaltene concentration, catalyst concentration and temperature, were investigated. Maximum adsorption occurred in the presence of 0.7 g L^−1^ of the adsorbent, at a concentration of 2000 mg L^−1^ of asphaltene. The asphaltene adsorption by NPs follows a quasi-second order adsorption kinetics. Asphaltene adsorption kinetics were studied by Langmuir, Freundlich, and Temkin isotherms. The prominent advantage of the adsorbent is its ability to be recovered after each adsorption by acid treatment, so that no significant reduction in adsorbent adsorption activity was observed, which can be directly attributed to the presence of ZrO_2_/SO_4_^2−^ groups in the hybrid.

## Abstract 2

A new, efficient and recyclable hybrid based on immobilized sulfated zirconium oxide on magnetite fibrous silica (KCC-1) has been developed and utilized for the efficient adsorption and removal of asphaltene from crude oil.

## Graphical abstract

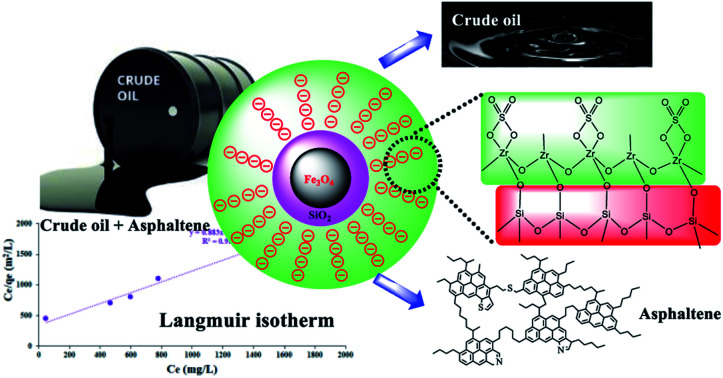

## Introduction

Sedimentation of heavy hydrocarbon materials includes two groups of asphaltene and wax. The closure of wells and process transmission lines, the failure of process equipment for operation, as well as a severe decline in catalyst efficiency are among the problems of sediment formation. In the face of the problem of asphaltene deposition in upstream industries, two strategies have been considered to prevent sediment formation and descaling treatment.^1,2^ In the process modification method, in addition to thermodynamic equilibrium conditions, fluid retention time is one of the effective factors in creating sediment. Chemical treatment, external force application, mechanical treatment, heat treatment and biotechnology method are among the methods of asphaltene removal in the production and operation processes. There are four methods to remove asphaltene from the bottom products of the vacuum distillation tower, which are solvent asphalting, asphalt oxidation, supercritical extraction and asphalt emulsion.^3^

**Scheme 1:**
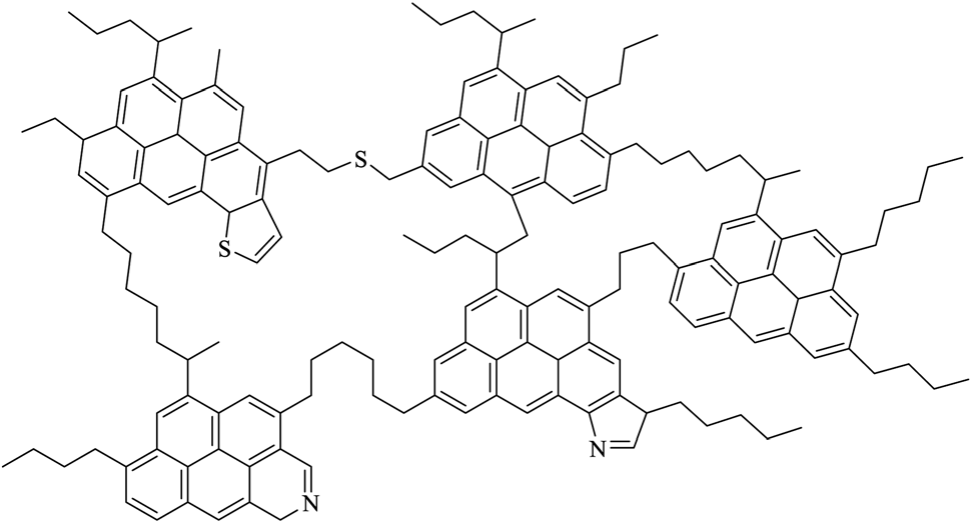
Structure of a possible asphaltene molecule.

Asphaltene (Scheme 1) is a section of crude oil or other carbonated source. Asphaltene contains complex molecules, which dissolve in toluene or dichloromethane but are insoluble in low boiling point paraffin solvents, such as normal heptane. Asphaltenes can be derived from crude oil, coal or oil shale. They are a polycystic cluster structure that has been alternately replaced by alkyl groups, and has heteroatoms (oxygen, nitrogen, sulfur) and very small amounts of metal (such as nickel and iron).^4^ The molecular formula of C_74_H_87_NS_2_O is proposed for the structure of a medium asphaltene. However, the exact molecular formula of asphaltene is unclear and its structure varies depending on its source. Scheme 1 shows a schematic of the asphaltene structure.^5,6^

**Fig. 1:**
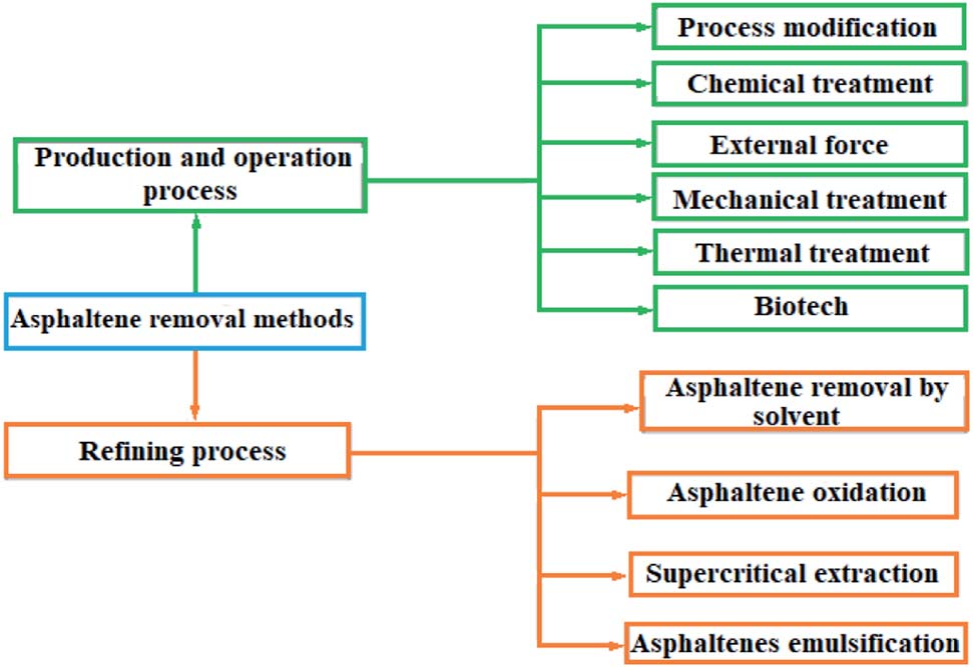
Asphaltene removal methods.

Various factors are effective in the formation and deposition of heavy organic materials from crude oil, which can be pointed to the characteristics and composition of the percentage of crude oil, the type of fluid injected, pressure, temperature, and the characteristics of canals (*e.g.*, well pipes) that the reservoir fluid flows through.^7^ The presence of asphaltene in crude oil has many problems, so that its non-removal from crude oil causes great and irreparable damage to the petrochemical industry. Hence, a wide variety of methods have been developed to remove asphaltene. Asphaltene removal methods are schematically shown in Fig. 1.

One of the effective methods to control asphaltene deposition is the use of chemical additives, such as surfactants,^3^ polymeric inhibitors^6^ and adsorbents. So far, several adsorbents for the adsorption of asphaltene from a petroleum solution have been developed, such as β-zeolite,^2^ minerals (kaolin, calcite, and dolomite), clay and source rock, and various types of metal oxide nanoparticles, such as MgO, TiO_2_, NiO, CaO, Fe_3_O_4_, and Al_2_O.^8^ The high surface-to-volume ratio and smaller size of NPs make them easier to use in porous media, and improves the fluid flow performance in oil extraction.^9,10^

The amount of adsorbed asphaltene on nanoparticles strongly depends on the type of surface chemistry of the NPs, and the type of interaction force between the asphaltene and the nanoparticles. Different types of forces for asphaltene adsorption on nanoparticles mainly include asphaltene surface charge, van der Waals force and acid–base interaction between the nanoparticle surface and polar asphaltene particles.^11^

Previous studies have shown that these nanoparticles are good options for the adsorption and catalytic oxidation of asphaltene in the process of improving the properties of crude oil. However, the difference in the adsorption rate is due to the nature and different properties of the nanoparticles. The functional groups in asphaltene make it possible for nanoparticles to absorb electrons by sharing them with asphaltene molecules at their surface.^8^ Various predictions have been made for the asphaltene adsorption on nanoparticles. Several researchers have predicted the presence of oxygen and nitrogen as an important factor in the asphaltene adsorption on nanoparticles. It has been found that many factors, such as the nitrogen content, water content and H/C ratio (or aromatic rings), affect the amount of adsorption. There have been several reports of metallic nanoparticles for asphaltene removal. Franco *et al.*^12^ tested 12 different types of nanoparticles for asphaltene removal. The results showed that asphaltene adsorption has a downward trend for AlNi15, SNi15, SNi5, AlNi5, silica gel (amorphous), silica gel (crystalline), Al(ii), zeolite, PdNi/Al, Al(i), and commercial silica gel. Nassar *et al.* showed that Ni–Pd bimetallic nanoparticles containing oxide substrates, such as SiO_2_, Al_2_O_3_ and TiO_2_, have higher adsorption capacity and catalytic activity toward asphaltene adsorption than nanoparticles that do not have oxide substrate and can be easily decomposed after adsorption.^13^ Magnetic metal oxides have also shown significant adsorption to asphaltene. Setoodeh *et al.* studied the close relationship between magnetic properties and the phenomenon of adsorption, and stated that the adsorption in paramagnetic compounds is much greater.^14^ The advantage of these compounds is their easy recovery after adsorption by an external magnetic field.

Due to their high specific surface area and high porosity, mesoporous materials show high surface activity, which has led to their widespread use as adsorbents, catalyst substrates and catalysts.^15^ The porous microstructure of these materials allows the creation of active catalytic sites on a large internal surface of porosities. This phenomenon leads to improved catalytic activity of the system. However, poor access to these active sites within the pores limits their use in cases where high mass transfer is required.^16^ Types of silica mesoporous materials include HMM (Hiroshima mesoporous materials), FSM (folded sheet mesoporous silica), MSU (MSU represents Michigan State University), KSW, SBA-15 (Santa Barbara Amorphous), MCM-41 (Mobil Composition of Matter) and KCC-1 (KCC represents KAUST Catalysis Centre), which have different shapes of porosities and sizes depending on the method of preparation. Among them, KCC-1 has excellent properties such as high specific surface area, morphology, fibrous surface, high mechanical stability and good thermal and hydrothermal stability, and is introduced as the best material for a catalyst substrate. KCC-1 was first invented in 2010 by Polshettiwar *et al.* as a new member of the mesoporous silica materials (MSMs) family in the KAUST Catalysis Centre.^16^ The fibrous silica KCC-1, like all members of the MSMs family, was synthesized by surfactant and silica molding, followed by mold removal. KCC-1 had a high surface area and pore volume due to the presence of fibrous silica and meso- and micro-porosities on these fibers. Polshettiwar reported that the large surface area of the material is due to the presence of dendrimeric fibrous silica and their associated channels, which in turn makes KCC-1 the first of its kind.^16^ It is also a good option for use in drug release, hydrogen storage and in the preparation of nanocomposites. Modification of the surface of mesoporous materials with minerals can change their properties, and improve their use for catalytic application or adsorption of certain compounds.^15,16^

Sulfated zirconium oxide is one of the compounds that has unique properties, such as high thermal stability, high chemical resistance, good catalytic activity and flexible modifiability, so that it has become a reliable candidate in catalytic applications. Limited reports of zirconium oxide are available for the adsorption and removal of organic/inorganic compounds. Previously, Pan *et al.* used a nano-hydrated zirconium oxide/polystyrene hybrid adsorbent for the removal of arsenic from water.^17^ Zirconium oxide-modified biochar for the adsorption of sulfate from water,^18^ hydrous zirconium oxide-based nanocomposite for the removal of phosphate from water,^19^ and zirconium sulfate-surfactant micelle mesostructure immobilized on a polymer matrix for the removal of phosphate from water^20^ are some of the applications of the zirconium oxide hybrids for adsorption goals. Ciesla and colleagues studied the potential of zirconium oxide as a precursor for the preparation of porous hybrids. Although its adsorption activity on a specific compound was not examined in this study, they showed that the resulting hybrid has high porosity and has high thermal stability.^21^ Therefore, modification of the KCC-1 surface with sulfated zirconium oxide is expected to improve its properties in order to adsorb and remove asphaltene. However, in a study on iron oxide, Nassar *et al.* showed that in the presence of iron oxide nanoparticles along with asphaltene, a significant reduction in the average activation energy is observed, which indicates the high catalytic activity of iron oxide. They concluded that iron oxide nanoparticles could be an excellent adsorbent or catalyst to improve the quality of heavy oil.^22^ In this work, magnetic Fe_3_O_4_ nanoparticles will be used to adsorb and remove asphaltene from oil. First, Fe_3_O_4_ nanoparticles were coated by silica groups, and then the KCC-1 shell is formed *in situ* on Fe_3_O_4_@SiO_2_ NPs. ZrO_2_/SO_4_^2−^ groups were then immobilized on silicate fibers in KCC-1, and the resulting Fe_3_O_4_@SiO_2_/KCC-1@ZrO_2_/SO_4_^2−^ nanoparticles were then used as a strong and recoverable adsorbent to remove asphaltene. The high acid stability of sulfated zirconium oxide groups in an acidic environment not only causes high adsorption stability during successive cycles, but also causes the adsorbent to maintain its adsorption activity after acid treatment.

## Experimental

### Materials

A sample of crude oil extracted from East Asaluyeh oil refinery, located in Asaluyeh (in Bandar Bushehr, IRAN), was investigated. Normal hexane, normal heptane and toluene with 99% purity were prepared by Merck, and used as received. Filter paper of 0.22 μm was used for asphaltene filtration. Other materials were provided from Merck and used as received. Solvents used were of analytical grade, and dried before use.

### Instrumentation

FT-IR spectra were obtained on a JASCO FT/IR 4600 spectrophotometer using a KBr disk. Field emission scanning electron microscopy (FE-SEM) images were taken using an FEI Quanta 200 apparatus. Energy dispersive X-ray (EDX) spectroscopy analyses were conducted on a JEOL 7600F FE-SEM instrument equipped with an energy dispersion X-ray spectrometer from Oxford Instruments. Transmission electron spectroscopy (TEM) images were taken using a Philips EM208S microscope operated at 100 kV. Magnetic behavior of the NPs was studied by vibrating sample magnetometer (VSM) method using a Lake Shore Cryotronics 7407 at room temperature. UV-Vis analyses were recorded with a SPECORD 210 PLUS Analytikjena spectrophotometer. Crystal structures of the samples were studied by X-ray diffraction (XRD) method with a Bruker D8/Advance powder X-ray diffractometer. The surface area, pore diameter, and pore volume of the samples were studied by N_2_ physisorption method at −196 °C with a Micromeritics ASAP 2000 instrument, surface area and pore size analyzer, using the BET (Brunauer–Emmett–Teller) method.

### Methods

#### Preparation of sulfated-zirconium oxide (ZrO_2_/SO_4_^2−^)

**Scheme 2:**
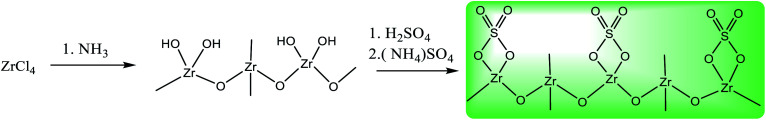
Preparation of sulfated-zirconium oxide from the ZrCl_4_ precursor.^23,24^

Sulfated zirconium oxide was prepared according to a previously reported procedure.^23,24^ First, to a solution of 2.3 g of ZrCl_4_ (as a precursor) in distilled water (10.0 mL), 10.0 mL of 1.0 N ammonia solution was added dropwise during a span of 30 min to the solution until the solution pH was adjusted to 11.0. The milky solution was aged for a day at room temperature. The resulting product (white powder) was washed by deionized water for five times (each time by 10.0 mL) using centrifugation until the solution pH reached 7.0. Then, the product (Zr(OH)_4_) was dried at 100 °C overnight (97% isolated yield, 1.6 g). To prepare ZrO_2_/SO_4_^2−^, 1.0 g of Zr(OH)_4_ was added to an aqueous (NH_4_)_2_SO_4_ (2.5 g) solution in 50 mL of distilled water with a 1 : 3 mixing ratio of Zr : S. The mixture was aged for 12 h at 80 °C, followed by calcination at 400 °C (10 °C) for 3 h under air atmosphere. The resulting ZrO_2_/SO_4_^2−^ was washed with deionized water three times (each by 10 mL), and then dried at 50 °C (Scheme 2).

#### Preparation of Fe_3_O_4_@SiO_2_/KCC-1

Fe_3_O_4_@SiO_2_/KCC-1 NPs was prepared according to a reported protocol elsewhere.^25,26^ Briefly, in the first step, a mixture of as-prepared Fe_3_O_4_ NPs (0.1 g) and urea (1.8 g) in 20 mL of distilled water was sonicated for 30 min at room temperature. Cetyl trimethyl ammonium bromide (CTAB, 1.0), *n*-butyl alcohol (1.0 g), and cyclohexane (20.0 g) were each added to the mixture. The mixture was stirred for 30 min at room temperature. Then, 1.0 g of tetraethyl orthosilicate (TEOS) was added dropwise to the mixture during a span of 10 min, and stirred by a mechanical stirrer for a day at a constant temperature of 70 °C. The product was separated by centrifugation, washed with cool EtOH and water (in order to further process), and then dried in a vacuum oven for 12 h (80 °C). Finally, Fe_3_O_4_@SiO_2_/KCC-1 NPs were prepared by removal of CTAB as the template *via* calcination of the product at 550 °C for 5 h under air atmosphere.

#### Preparation of Fe_3_O_4_@SiO_2_/KCC-1@ZrO_2_/SO_4_^2−^

**Scheme 3:**
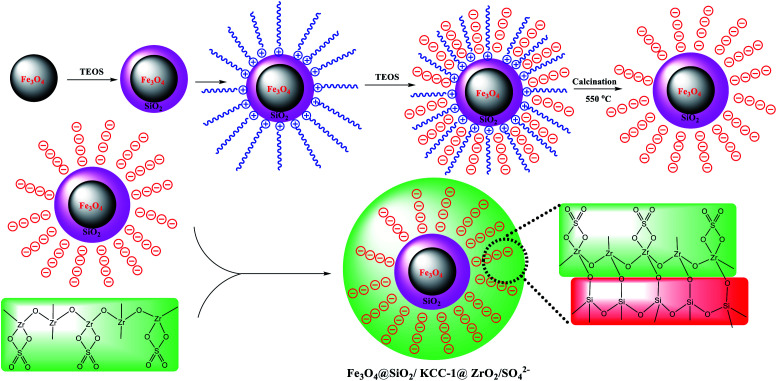
A general schematic view for the preparation of Fe_3_O_4_@SiO_2_ KCC-1@ZrO_2_/SO_4_^2−^ (this is a proposed structure for Fe_3_O_4_@SiO_2_/KCC-1@ZrO_2_/SO_4_^2−^ based on the characterization analyses results).

Functionalization of Fe_3_O_4_@SiO_2_/KCC-1 with ZrO_2_/SO_4_^2−^ was performed based on a procedure reported elsewhere with slight modifications.^27^ For this goal, 0.2 g of Fe_3_O_4_@SiO_2_/KCC-1 was dispersed in H_2_O : EtOH solution (15 mL, 1 : 3, v/v) for 15 min. Separately, ZrO_2_/SO_4_^2−^ (0.4 g) was dispersed in EtOH (10 mL), and added dropwise to the first solution that underwent sonication during a span of 10 min at room temperature. Then, 15 mL of 10% w/w NaOH was added dropwise to the solution and stirred for 24 h. The obtained Fe_3_O_4_@SiO_2_/KCC-1@ZrO_2_/SO_4_^2−^ was separated from the mixture by applying an external magnetic field, then isolated at room temperature after washing by deionized water and drying for 12 h at 50 °C. Scheme 3 shows a schematic view for this preparation.

#### Preparation of KCC-1 nanoparticles (used in control tests)

Pure KCC-1 nanoparticles were prepared by simple sol–gel method under hydrothermal conditions in a stainless-steel autoclave containing a Teflon chamber.^28^ In this method, a combination of urea (2.4 g), CTAB (2.5 g), and deionized water (250 mL) was added to a 500 mL flask. The mixture was stirred at room temperature for 20 minutes. Then, a homogeneous mixture of 12.5 g TEOS in 250 mL of cyclohexane was added dropwise to the reaction mixture for 25 minutes, and the original mixture was stirred at room temperature for 30 minutes to obtain a milky solution. The resulting mixture was transferred to a one-liter autoclave with a Teflon chamber. The autoclave was then placed into a furnace at 400 °C for 8 hours. The autoclave was then allowed to cool to room temperature. Then, the gel-like white product was separated by centrifugation at 6000 rpm, and washed with water and ethanol (three times in 50 mL volumes each) to separate impurities and unreacted materials. The product was dried overnight at 363 °C and finally placed in a furnace at 823 °C for 6 hours with the aim of calcination. The resulting KCC-1 nanoparticles were stored in a sterile container with a white powdery appearance.

#### Re-activation of adsorbents

**Scheme 4:**
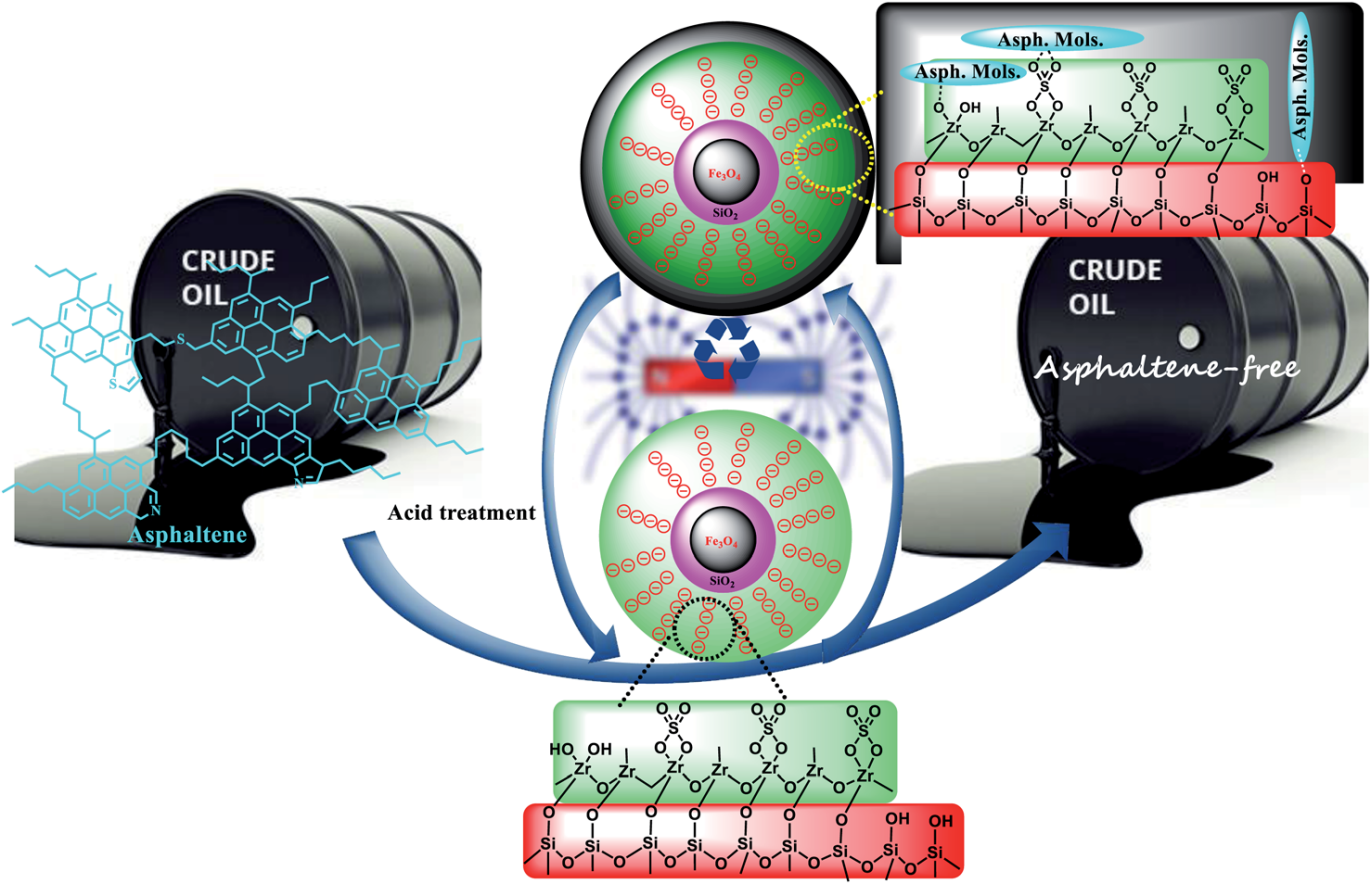
Adsorption of asphaltene on Fe_3_O_4_@SiO_2_/KCC-1@ZrO_2_/SO_4_^2−^ recyclable NPs.

Recyclability of the adsorbents was studied in 5 consecutive cycles. For this purpose, the adsorbent was separated from the reaction mixture after each run by applying an external magnetic field, washed with deionized water and treated with 10 mL of 1 N HCl. The catalyst was stirred for 2 hours in an acidic solution at 50 °C, removed by applying an external magnetic field, and then washed with deionized water. Finally, the nanoparticles were dried at 150 °C for 8 hours and reused. Drying of nanoparticles at this temperature was performed to remove the surface water of the catalyst and increase the adsorption efficiency of asphaltene (Scheme 4).^8^

#### Asphaltene extraction

The standard method of ASTM D6560 (IP-143) was used to determine the amount of asphaltene in crude oil.^29^ Two types of normal paraffin (normal hexane and normal pentane), and toluene were used to precipitate and dissolve asphaltene, respectively. Normal heptane was used to extract the maximum amount of asphaltene in crude oil due to its highest dissolution power among all normal paraffins. Initially, 2.5 g of crude oil was weighed and added to 100 g of normal paraffin. Primary reflux was performed to precipitate the asphaltene in the crude oil using normal paraffin in a Soxhlet extractor. The time of the first reflux is 2 hours, and the normal paraffin-crude oil solution was then placed overnight under dark conditions. In the next step, the solution was filtered using a filter paper (Whatman Grade 42 circles, diam. 42.5 mm, ashless). Asphaltene and a small amount of other components in the crude oil accumulate on the filter paper, and the other three components (saturated and aromatic compounds and resin) pass through the filter paper. To separate the other adsorbed components on the filter (except asphaltene), 50 mL of normal heptane (or normal hexane) is poured into the balloon. The second reflux is performed until the filter is completely black, which is related to asphaltene.

In order to separate asphaltene from the filter paper, 70 mL of toluene was poured into the balloon. The third reflux was performed until the filter paper returned to its original color. The solution was poured into a weighed cylinder, and placed at room temperature to evaporate the solvent. After 24 hours, the mass of the cylinder was measured.

#### Calibration curve

In order to measure the interfacial properties of asphaltene, a variety of pure asphaltene in the range of 50–2000 mg L^−1^ extracted by IP-143 test was prepared in toluene. The absorption of the solutions was recorded in *λ*_max_ related to asphaltene at 300 nm^28–33^ by UV-Vis spectrophotometer.

#### Asphalt removal study

To study the removal of asphaltene, a certain amount of adsorbents was added to 10 mL of standard solutions prepared from asphaltene (250–2000 mg L^−1^). The solutions were then stirred for 12 h at room temperature. Fe_3_O_4_@SiO_2_/KCC-1@ZrO_2_/SO_4_^2−^ was separated from the reaction mixture by applying an external magnetic field (Scheme 4). Then, the absorbance of the solutions was recorded by UV-Vis. The equilibrium concentration (*C*_e_) of asphaltene can be obtained after the addition of each of the adsorbents. The percentage of asphaltene removal by the adsorbents was calculated by eqn (1):^33^1
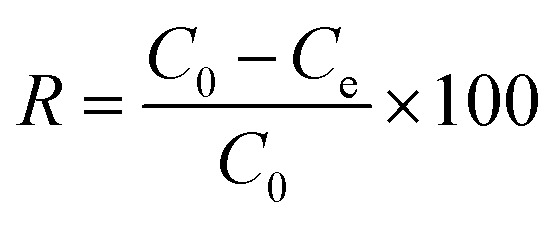
where *R* is the percentage of asphaltene removed from the solution, C_0_ and *C*_e_ are the initial concentration and the equilibrium concentration of asphaltene in mg L^−1^, respectively. The adsorption capacity of asphaltene by the adsorbents was calculated by eqn (2):^33^2
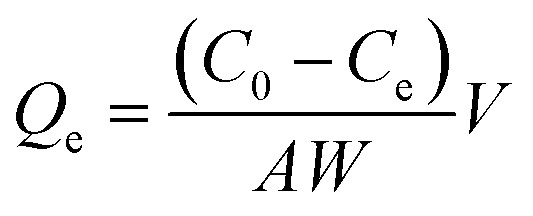
where *V* is the volume of solution (in L), *W* is the weight of the adsorbent (in g), *A* is the specific surface area in m^2^ g^−1^ and *Q*_e_ is the final adsorbent capacity in mg of asphaltene per gram of adsorbent mg m^−1^.^2^

## Results and discussion

### Catalyst characterization

**Fig. 2:**
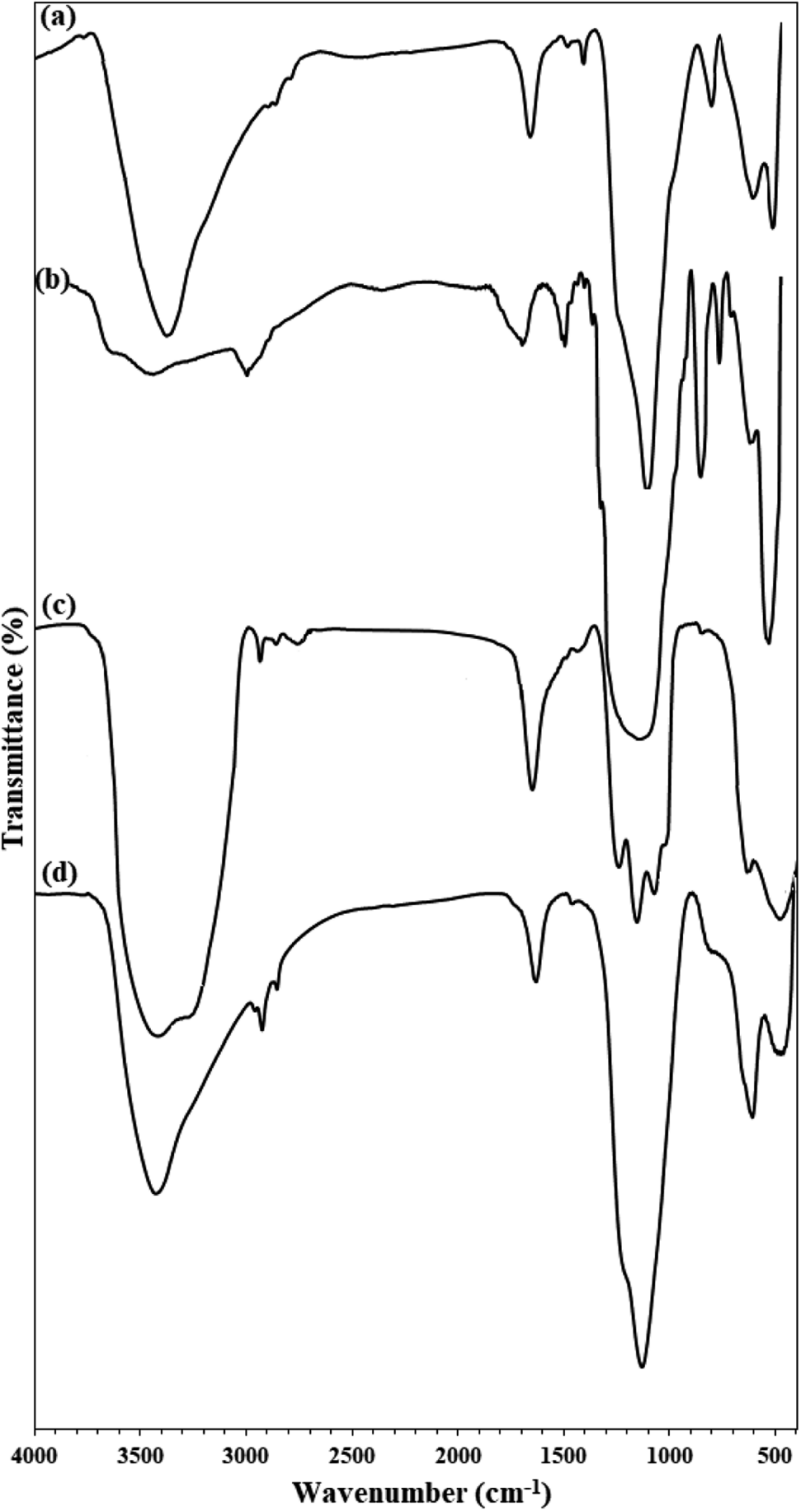
FTIR spectra of (a) Fe_3_O_4_@SiO_2_, (b) Fe_3_O_4_@KCC-1, (c) ZrO_2_/SO_4_^2−^, (d) Fe_3_O_4_@SiO_2_/KCC-1@ZrO_2_/SO_4_^2−^.

Fe_3_O_4_@KCC-1 and Fe_3_O_4_@SiO_2_/KCC-1@ZrO_2_/SO_4_^2−^ NPs were studied by different characterization methods. Fig. 2 shows the FTIR spectra of Fe_3_O_4_@SiO_2_, Fe_3_O_4_@KCC-1, ZrO_2_/SO_4_^2−^, and Fe_3_O_4_@SiO_2_/KCC-1@ZrO_2_/SO_4_^2−^ NPs. Two characteristic peaks at 1100 and 800 cm^−1^ represent the Si–O–Si asymmetrical and symmetrical stretching vibrations, respectively, for the Fe_3_O_4_@SiO_2_ NPs. The presence of a characteristic peak at 560 cm^−1^ related to Fe–O vibrations (Str.) represents the incorporation of Fe_3_O_4_ nanoparticles within the Fe_3_O_4_@SiO_2_ framework.^34^Fig. 2b shows the vibrations related to KCC-1 nanoparticles by two characteristic peaks at 1030, 780 cm^−1^, respectively, related to symmetric and asymmetric Si–O–Si vibrations, respectively, in full agreement with the literature.^35^ Sulfated zirconium oxide (ZrO_2_/SO_4_^2−^) was characterized by three characteristic peaks at 1142, 1045 cm^−1^, and a shoulder at 994 cm^−1^ related to the asymmetric and symmetric modes of the S=O or S–O stretching vibrations coordinated to a metal cation.^36^ The series of peaks appearing at 466–748 cm^−1^ were attributed to the Zr–O–Zr asymmetric stretching vibrations (Fig. 2c).^37^ In addition, two peaks at 3420 cm^−1^ (broad) and 1636 cm^−1^ (medium) represent the stretching and bending vibrations, respectively, related to the adsorbed (or coordinated) water molecules into sulfate groups in ZrO_2_/SO_4_^2−^ in agreement with the literature (Fig. 2c).^38^ The immobilization of ZrO_2_/SO_4_^2−^ on Fe_3_O_4_@SiO_2_/KCC-1 caused the formation of a new peak at 802 cm^−1^ related to Zr–O–Si stretching vibration (Fig. 2d).^34^ In addition, all vibrations related to both ZrO_2_/SO_4_^2−^ and Fe_3_O_4_@KCC-1 could be found in the FTIR spectrum of Fe_3_O_4_@SiO_2_/KCC-1@ZrO_2_/SO_4_^2−^ (Fig. 2d).

**Fig. 3:**
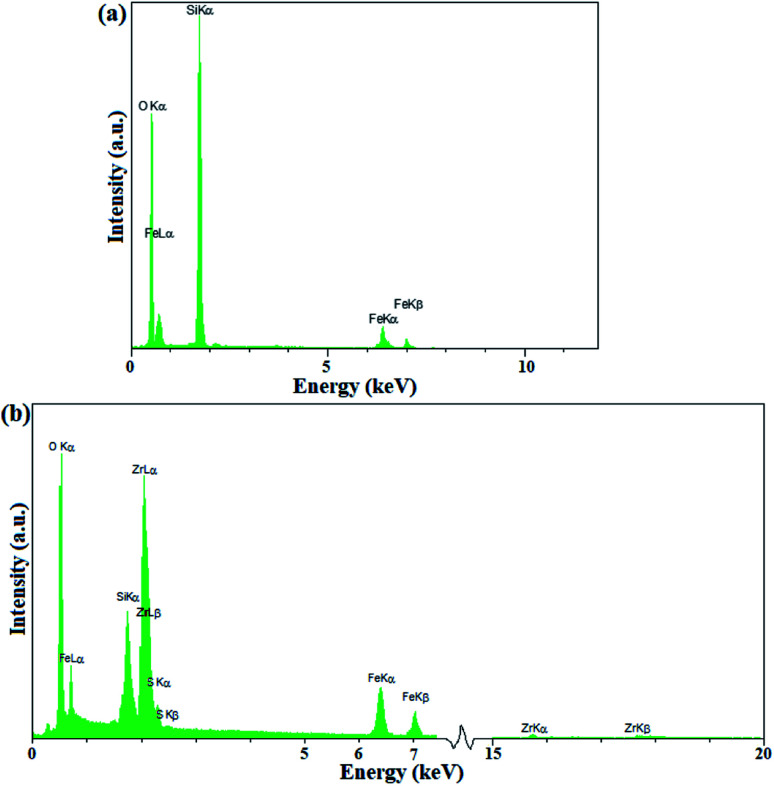
EDX spectra of (a) Fe_3_O_4_@KCC-1 and (b) Fe_3_O_4_@SiO_2_/KCC-1@ZrO_2_/SO_4_^2−^.

The presence of elements in Fe_3_O_4_@KCC-1 and Fe_3_O_4_@SiO_2_/KCC-1@ZrO_2_/SO_4_^2−^ was investigated by EDX analysis. As shown in Fig. 3a, Fe_3_O_4_@KCC-1 shows three elements of O, Fe, Si, which is in agreement with the previously reported literature.^35^ The EDX spectrum of Fe_3_O_4_@SiO_2_/KCC-1@ZrO_2_/SO_4_^2−^ also confirmed its structure by detection of all expected elements, including O, Fe, Zr, Si, and S. More importantly, the absence of any additional peaks in the EDX spectra of Fe_3_O_4_@KCC-1 and Fe_3_O_4_@SiO_2_/KCC-1@ZrO_2_/SO_4_^2−^ indicates the high purity of the prepared compounds.

The crystal structure of the nanoparticles was studied by X-ray diffraction analysis. The X-ray diffraction pattern of ZrO_2_/SO_4_^2−^ fully confirms its structure according to previously reported structures.^27,36^

**Fig. 4:**
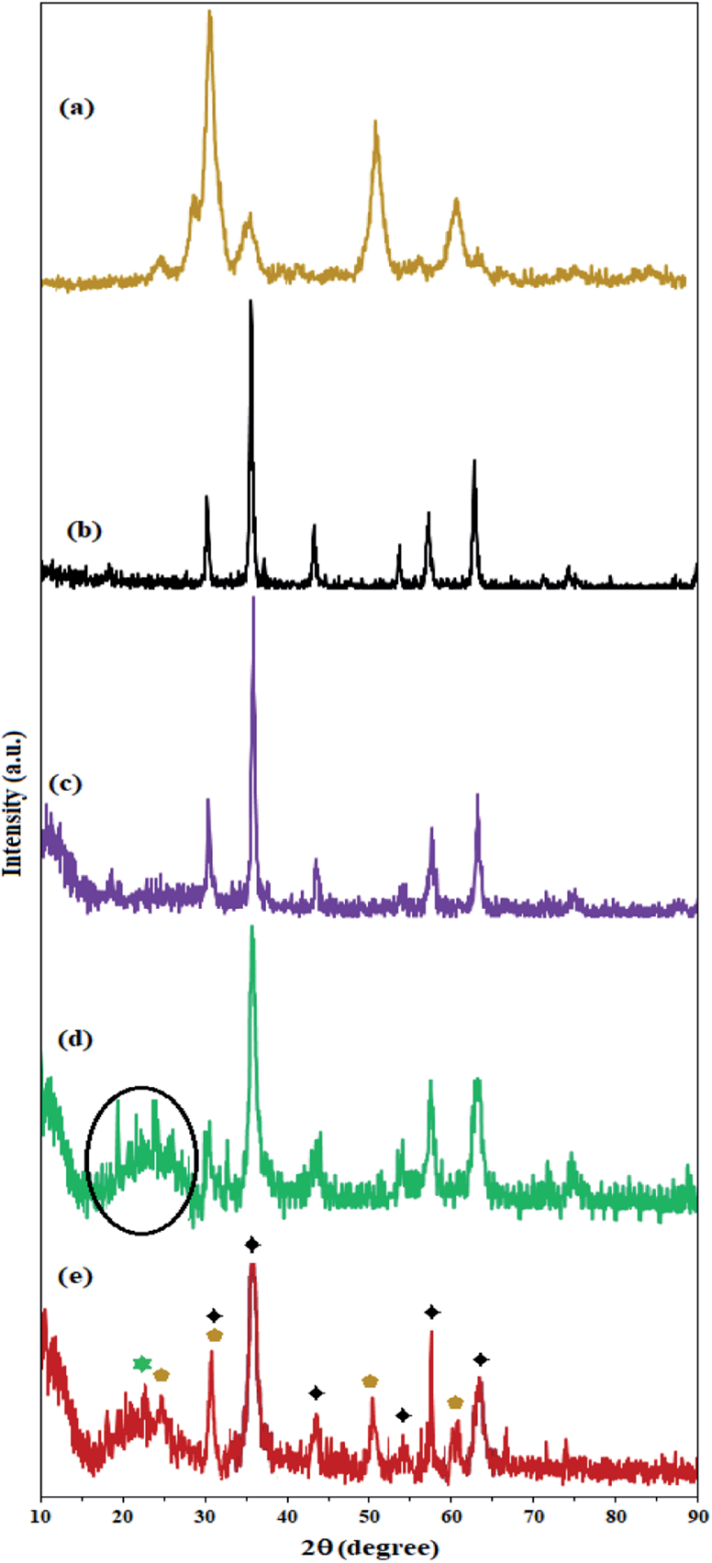
XRD patterns of (a) ZrO_2_/SO_4_^2−^, (b) Fe_3_O_4_, (c) Fe_3_O_4_@SiO_2_, (d) Fe_3_O_4_@KCC-1, and (e) Fe_3_O_4_@SiO_2_/KCC-1@ZrO_2_/SO_4_^2−^.

As shown in Fig. 4a, the zirconium X-ray diffraction pattern has 3 characteristic peaks at 2*θ* = 30.5°, 50.3°, and 60.1°, which are in accordance with the ZrO_2_/SO_4_^2−^ high-crystalline tetragonal structure (JCPDS 17-0923).^36,39^ The crystal structure of Fe_3_O_4_ NPs is completely in line with the X-ray diffraction pattern of the cubic phase of Fe_3_O_4_ particles (Fig. 4b).^40,41^ Six characteristic peaks at 2*θ* = 30.0°, 35.5°, 43.1°, 53.7°, 57.1°, and 62.5° corresponding to the planes (indices) (220), (311), (400), (422), (511), and (440) respectively, were exactly in line with the known X-ray diffraction pattern for Fe_3_O_4_ NPs (reference JCPDS card no. 19-629).^34^ The coating of these nanoparticles with amorphous silicate groups reduced the crystallinity of the peaks and also caused the appearance of an amorphous peak at 2*θ* = 12.5°, which confirms the successful coating of the nanoparticles by silicate groups (Fig. 4c).^34^ The immobilization of KCC-1 groups on Fe_3_O_4_@SiO_2_ NPs was also confirmed by the broad amorphous peak at 2*θ* = 22.3° (specified in the spectrum) for the fibrous silicate in KCC-1 (Fig. 4d).^42^ The presence of peaks related to the crystal structure of Fe_3_O_4_ and SiO_2_ indicates that the crystal structure of the nanoparticles does not change during the functionalization process. Immobilization of the sulfated zirconium oxide groups on silicate fibers in KCC-1 produces characteristic peaks related to the crystal structure of ZrO_2_/SO_4_^2−^ (specified with cyclopentane markings) in the X-ray diffraction pattern of Fe_3_O_4_@SiO_2_/KCC-1@ZrO_2_/SO_4_^2−^. In addition, the crystal structure of Fe_3_O_4_ is well illustrated by maintaining its characteristic peaks (with little displacement) in the X-ray diffraction pattern of the absorber with black stars. The peak corresponding to the KCC-1 groups is also shown at 2*θ* = 22.5° with a green star (Fig. 4e).^43^

**Fig. 5:**
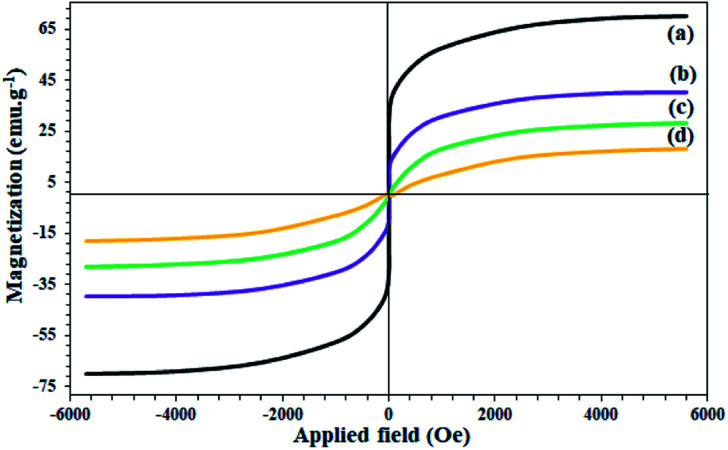
VSM curves of (a) Fe_3_O_4_, (b) Fe_3_O_4_@SiO_2_, (c) Fe_3_O_4_@KCC-1, and (d) Fe_3_O_4_@SiO_2_/KCC-1@ZrO_2_/SO_4_^2−^.

The study on the magnetic properties of nanoparticles by VSM method showed that all nanoparticles have superparamagnetic properties due to the magnetic behavior of Fe_3_O_4_ nanoparticles (with coercivity equal to zero). As shown in Fig. 5, the saturation magnetization of Fe_3_O_4_, Fe_3_O_4_@SiO_2_, Fe_3_O_4_@KCC-1, and Fe_3_O_4_@SiO_2_/KCC-1@ZrO_2_/SO_4_^2−^ NPs are equal to 70, 40, 28, and 18 emu g^−1^, respectively. The decrease in the amount of magnetization at each stage of the functionalization of Fe_3_O_4_ indicates the successful immobilization of different groups on it (Fig. 5b–d).

Despite the reduction of magnetization of Fe_3_O_4_-containing nanoparticles to 18 units, the nanoparticles were easily separated from the reaction mixture even after less asphaltene adsorption by applying an external magnetic field (Fig. 5d). The study on the thermal behavior of nanoparticles Fe_3_O_4_@SiO_2_, Fe_3_O_4_@KCC-1, and Fe_3_O_4_@SiO_2_/KCC-1@ZrO_2_/SO_4_^2−^ NPs showed that all of them have good stability and good correlation with other analyses, as well as their structures.

**Fig. 6:**
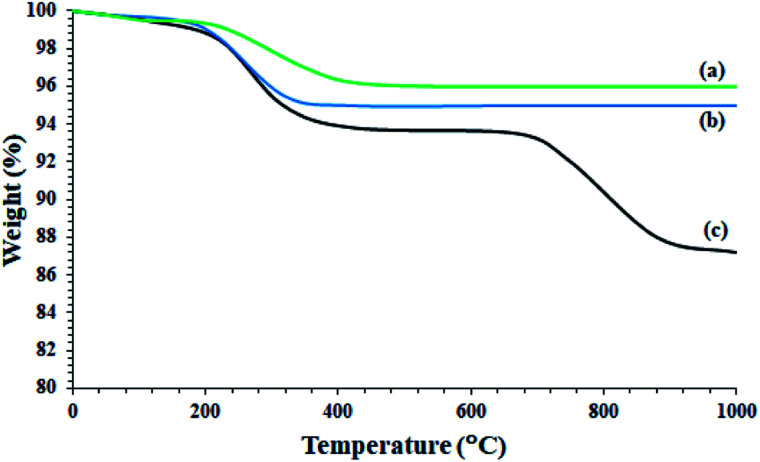
TGA curves of (a) Fe_3_O_4_@SiO_2_, (b) Fe_3_O_4_@KCC-1, and (c) Fe_3_O_4_@SiO_2_/KCC-1@ZrO_2_/SO_4_^2−^.

As shown in Fig. 6, Fe_3_O_4_@SiO_2_ NPs show only one peak at 220 °C in accordance with the 3% weight loss. This is consistent with the removal of water trapped in the nanoparticle structure, and is fully consistent with the literature (Fig. 6a).^34,41,44^ A slight weight loss below 220 °C was also attributed to the removal of adsorbed water (Fig. 6a).^34^ Fe_3_O_4_@KCC-1 nanoparticles also showed a weight loss of 5% at around 220 °C, and no further weight loss was observed up to 1000 °C (Fig. 6b). The Fe_3_O_4_@SiO_2_/KCC-1@ZrO_2_/SO_4_^2−^ NPs showed two weight losses at 220 °C and 750 °C, respectively, in accordance with the removal of trapped water and the removal/decomposition of sulfate groups on ZrO_2_/SO_4_^2−^.^27,36,39^ The presence of a peak at 750 °C with a weight loss of about 7% confirms the successful immobilization of ZrO_2_/SO_4_^2−^ groups on Fe_3_O_4_@KCC-1 NPs. In addition, the absence of any other weight loss peaks in the TGA spectrum of Fe_3_O_4_@SiO_2_/KCC-1@ZrO_2_/SO_4_^2−^ indicates high purity, as well as high stability of the hybrid.

**Fig. 7:**
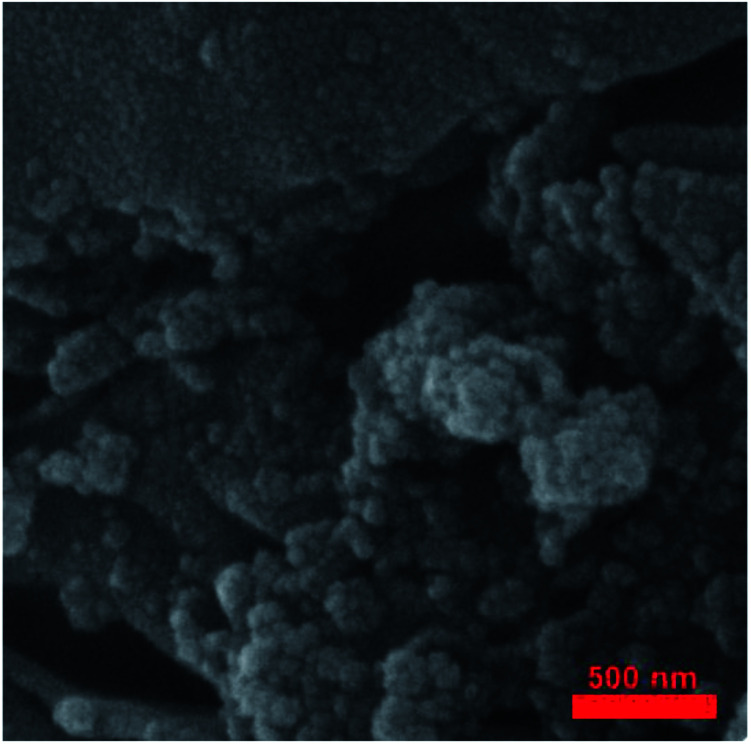
FE-SEM image of Fe_3_O_4_@SiO_2_/KCC-1@ZrO_2_/SO_4_^2−^.

Fig. 7 shows the FE-SEM image of Fe_3_O_4_@SiO_2_/KCC-1@ZrO_2_/SO_4_^2−^ nanoparticles. The nanoparticles have a homogeneous morphology according to previously reported SEM images.^27,36,39^

**Fig. 8:**
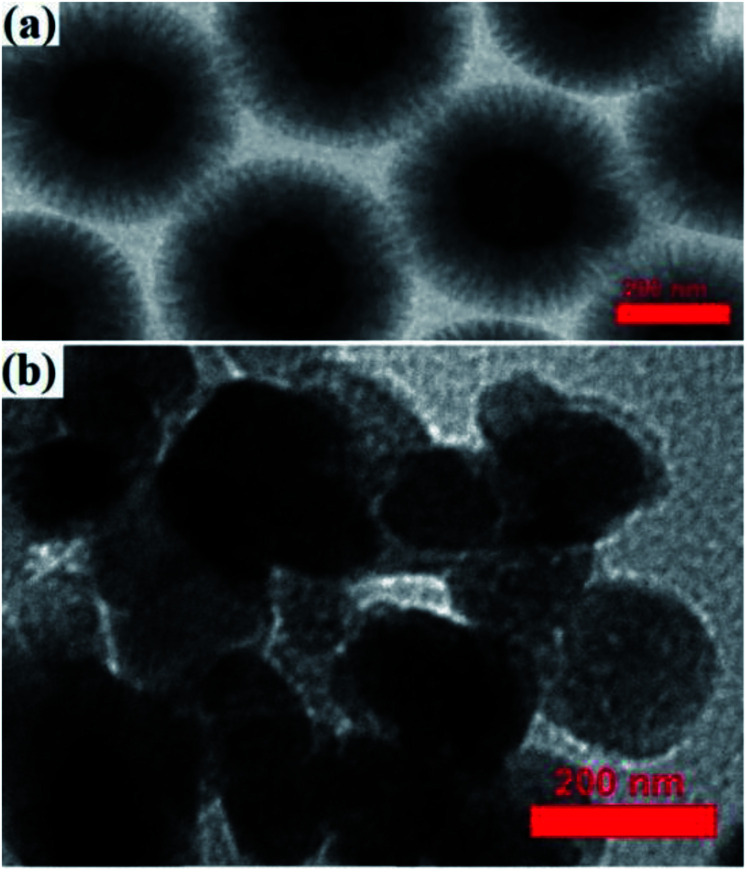
TEM images of (a) Fe_3_O_4_@KCC-1 and (b) Fe_3_O_4_@SiO_2_/KCC-1@ZrO_2_/SO_4_^2−^.

A TEM image of the Fe_3_O_4_@KCC-1 NPs showed that they have uniform fibers of silica that are growing from the center to the outside with a mean diameter of 200 nm and homogeneous morphology (Fig. 8a).

Immobilization of ZrO_2_/SO_4_^2−^ groups on silicate groups in Fe_3_O_4_@SiO_2_/KCC-1 caused a change in the particle morphology, according to what is shown in Fig. 8. As shown in Fig. 8, the particles have been coming out from the fibrous state of the silicate, which confirms the immobilization of the zirconium groups by the formation of Zr–O–Si bonds. The proposed structure for adsorbent NPs in Scheme 3 was also proposed in accordance with these results. The significant reduction in the crystal structure of the particles resulting from the analysis of X-ray diffraction of the adsorbent also confirms that the crystalline groups of ZrO_2_/SO_4_^2−^ are in contact with the fibrous and amorphous silicate.

Immobilization of porous sulfated zirconium oxide groups on the framework of Fe_3_O_4_@SiO_2_/KCC-1 and on silicate groups caused a significant increase in the porosity by 0.925 cm^3^ g^−1^ (Table 1). Significant reduction of the average porosity radius (1.992 nm) after immobilization of the sulfated zirconium oxide groups on silicate groups also reflects the successful immobilization of these groups (Table 1).

**Table 1:** Surface characteristics of ZrO_2_/SO_4_^2−^, Fe_3_O_4_, Fe_3_O_4_@SiO_2_, Fe_3_O_4_@KCC-1, and Fe_3_O_4_@SiO_2_/KCC-1@ZrO_2_/SO_4_^2−^

Entry	Sample	Specific surface area (m^2^ g^−1^)	Pore volume (cm^3^ g^−1^)	Average pore radius (nm)
1	ZrO_2_/SO_4_^2−^	126	0.392	6.571
2	Fe_3_O_4_	480	0.815	1.255
3	Fe_3_O_4_@SiO_2_	450	0.786	1.788
4	Fe_3_O_4_@KCC-1	370	0.520	6.652
5	Fe_3_O_4_@SiO_2_/KCC−1@ZrO_2_/SO_4_^2−^	120	0.925	1.992

This increase in porosity along with the increased acidity of the Fe_3_O_4_@SiO_2_/KCC-1@ZrO_2_/SO_4_^2−^ catalyst will increase the efficiency of the catalyst to adsorb asphaltene. As will be discussed in the next section, the presence of sulfated zirconium oxide groups in the catalyst results in greater stability and maintenance of the catalytic activity of the catalyst during successive cycles, while Fe_3_O_4_@KCC-1 nanoparticles suffer a decrease in catalytic activity during successive cycles. It can be attributed to the poisoning of catalytically active sites during successive cycles. In addition, a significant reduction of the specific surface area in the final catalyst indicates a successful and significant immobilization of sulfate groups on Fe_3_O_4_@KCC-1 nanoparticles.

### Asphaltene adsorption

#### Optimization of parameters involved in asphaltene adsorption from crude oil

##### Catalyst amount

**Fig. 9:**
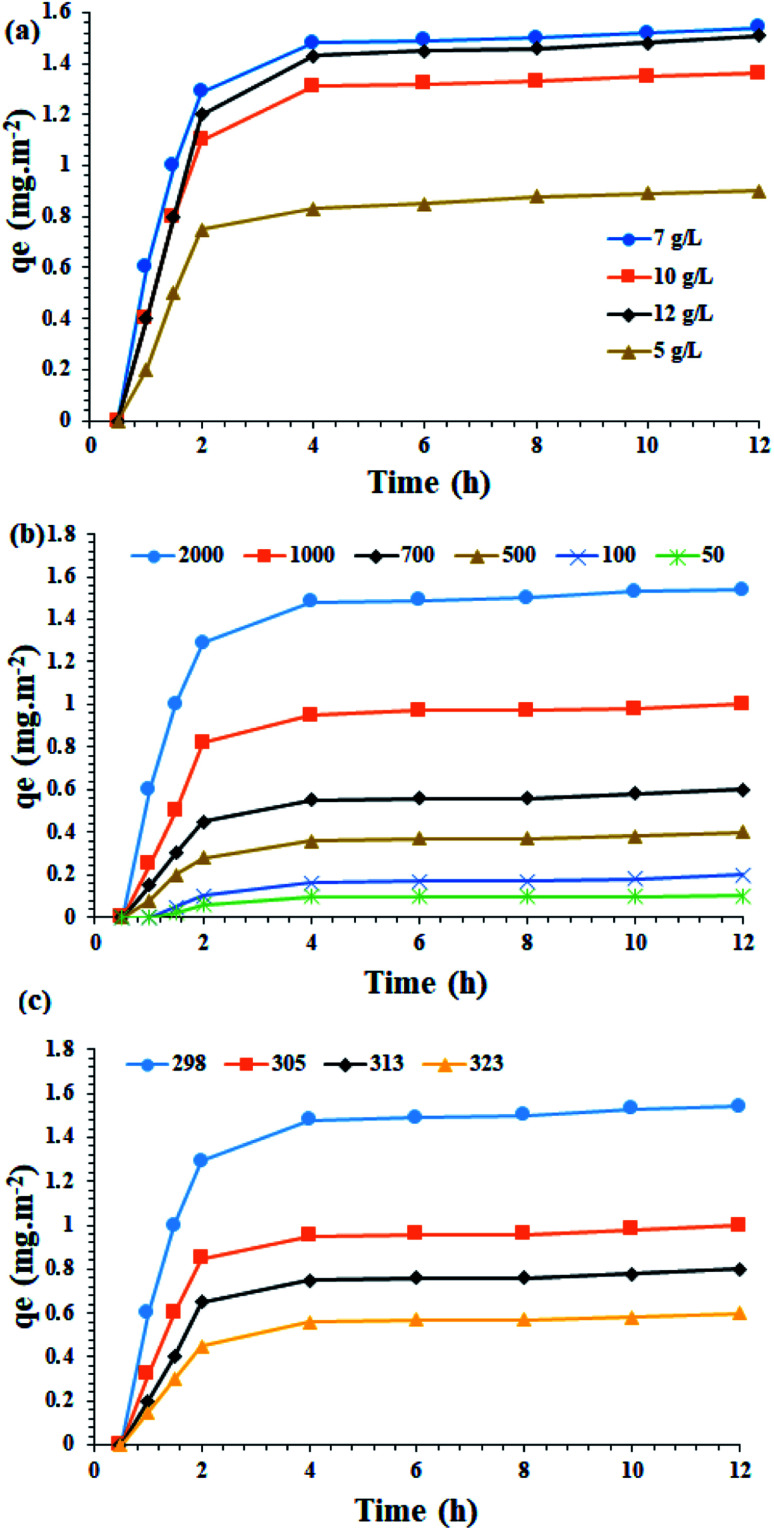
(a) Influence of the adsorbent amount (Fe_3_O_4_@SiO_2_/KCC-1@ZrO_2_/SO_4_^2−^) towards asphaltene adsorption at 298 K in the presence of 2000 mg L^−1^ of asphaltene. (b) Effect of the asphaltene concentration over the asphaltene adsorption on Fe_3_O_4_@SiO_2_/KCC-1@ZrO_2_/SO_4_^2−^ NPs (0.7 g L^−1^) at 298 K. (c) Influence of the reaction temperature over the asphaltene adsorption on Fe_3_O_4_@SiO_2_/KCC-1@ZrO_2_/SO_4_^2^ NPs (0.7 g L^−1^) in 2000 mg L^−1^ of asphaltene solution.

In the first step, the effect of different concentrations of Fe_3_O_4_@SiO_2_/KCC-1@ZrO_2_/SO_4_^2−^ on asphaltene adsorption was studied. As shown in Fig. 9a, the highest adsorption rates of 1.54 mg m^−2^ occurred in 0.7 g L^−1^ (in toluene) of the adsorbent.

##### Effect of the initial asphaltene concentration

In order to evaluate the effect of the initial asphaltene concentration, experiments with an initial asphaltene concentration of 50 to 2000 mg L^−1^ were performed over the Fe_3_O_4_@SiO_2_/KCC-1@ZrO_2_/SO_4_^2−^ adsorbent at a contact time of 12 h at 298 K. The results in Fig. 9b show that the initial concentration of aspartate has a significant effect on the rate of asphaltene adsorption by the absorbents. So, by increasing the initial concentration of asphaltene from 50 to 2000 mg L^−1^, the amount of asphaltene adsorption on both absorbents has increased. This could be due to the higher concentration gradient between the asphaltene solution and the adsorbent, which results in a stronger driving force for adsorption and consequently a higher adsorption capacity. As the initial concentration of asphaltene increases, the propulsive force of mass transfer increases, which in turn increases the amount of adsorption capacity.^28^ In other words, increasing the initial amount of adsorbent improves its propulsive force to penetrate or transfer this component in the thickness of the film layer, and also increases the adsorption speed.

This allows the adsorbent to reach its maximum capacity in the shortest possible time. The results obtained in this regard are in accordance with the results presented by the researchers.^28,30,45^ This study shows that at different temperatures, increasing the asphaltene concentration increases the adsorption on the adsorbent. Thus, after 2 h, the adsorbent is saturated and reaches its equilibrium adsorption capacity (Fig. 9b). At ambient temperature for 100 mg L^−1^, the experimental equilibrium adsorption *q*_e_ for the adsorbent was found to be 0.2 mg m^−2^. When the concentration increased to 2000 mg L^−1^, it reaches 1.54 mg m^−2^. The equilibrium adsorption rate of the adsorbent has a higher adsorption rate than materials, such as titanium oxide, magnesium oxide and calcium oxide, which indicates the suitability of these NPs for use as an asphaltene adsorbent. The results clearly show the strong adsorption capacity for Fe_3_O_4_@SiO_2_/KCC-1@ZrO_2_/SO_4_^2−^, which can be directly attributed to the presence of KCC-1, as well as ZrO_2_/SO_4_^2−^ groups immobilized on the Fe_3_O_4_@SiO_2_ framework. As will be shown in the control experiments, the strong adsorption on the Fe_3_O_4_@SiO_2_/KCC-1@ZrO_2_/SO_4_^2−^ NPs adsorbent could be attributed to the synergistic effect of the adsorbent groups KCC-1 and ZrO_2_/SO_4_^2−^, as each has a significant absorption of asphaltene. In addition, the presence of ZrO_2_/SO_4_^2−^ groups in a spherical structure and on silicate fibers caused the maximal adsorption of asphaltene.

##### Temperature effect

In the next step, the amount of adsorbed asphaltene at the optimal concentration of 2000 mg L^−1^ was examined at different temperatures of 298, 305, 313 and 323 K. An optimal concentration of 0.7 g L^−1^ of the adsorbent was utilized. Fig. 9c shows the results of the effect of temperature on asphaltene adsorption on Fe_3_O_4_@SiO_2_/KCC-1@ZrO_2_/SO_4_^2−^ NPs. As shown in Fig. 9c, when the temperature drops from 298 to 323 K, the adsorption capacity of the asphaltene on the adsorbent decreases. Therefore, it can be concluded that the lower temperature facilitates the adsorption of asphaltene on the adsorbent, completely in accordance with previous published papers.^28,30–32,46^

**Fig. 10:**
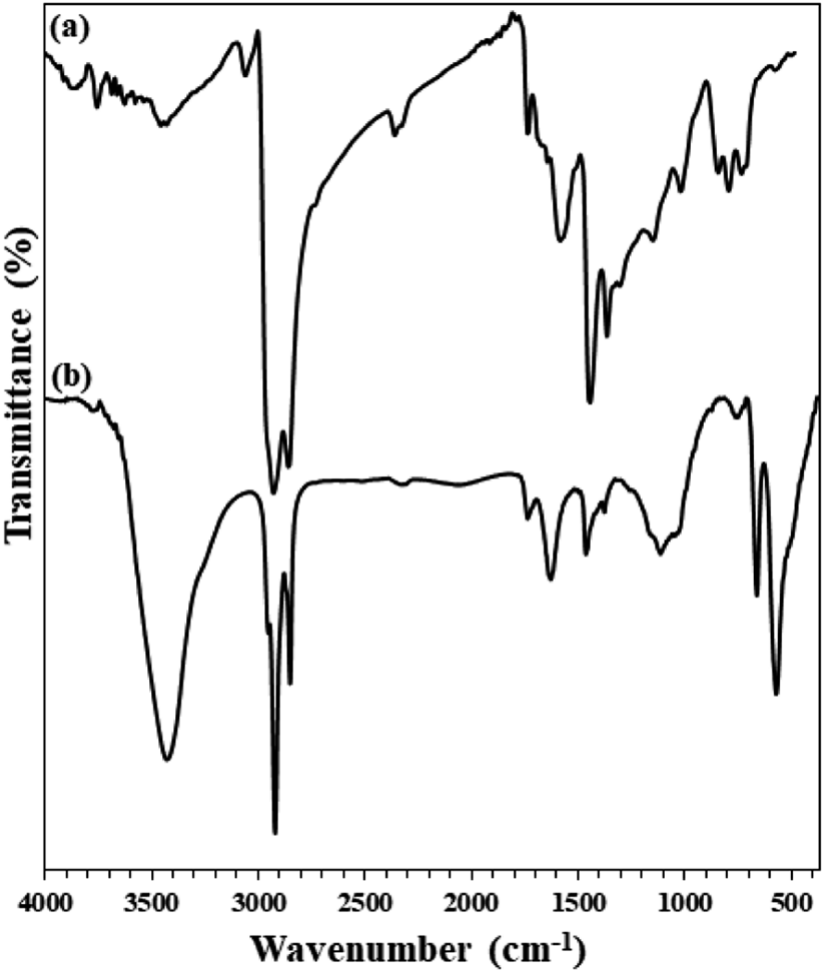
FTIR spectra of (a) asphaltene and (b) adsorbed asphaltene on Fe_3_O_4_@SiO_2_/KCC-1@ZrO_2_/SO_4_^2−^.

Therefore, kinetic studies at 2000 mg L^−1^ of asphaltene at optimal value of 0.7 g L^−1^ of Fe_3_O_4_@SiO_2_/KCC-1@ZrO_2_/SO_4_^2^ NPs adsorbent was studied. Asphaltene adsorption over Fe_3_O_4_@KCC-1 and over Fe_3_O_4_@SiO_2_/KCC-1@ZrO_2_/SO_4_^2−^ were studied by FTIR analysis. Asphaltene has a strong absorption related to the =C–H and –C–H stretching vibrations at 2900–3050 cm^−1^. A series of peaks appearing at 1580–1610 cm^−1^ were assigned to C=C bond stretching vibrations.^33^ In addition, a weak absorption at 1737 cm^−1^ was related to the C=O stretching vibration in carbonyl groups. A peak at 1160 cm^−1^ was assigned to ester/ether bonds in the asphaltene framework (Fig. 10). Fig. 10 shows the FTIR spectra of asphaltene adsorption by Fe_3_O_4_@KCC-1, and Fe_3_O_4_@SiO_2_/KCC-1@ZrO_2_/SO_4_^2−^. As shown in the figure, the presence of three characteristic peaks related to the structure of asphaltene in 2917, 2855 and 1452 cm^−1^ shows the physical adsorption of asphaltene on each of the nanoparticles.

### Adsorption isotherms

**Fig. 11:**
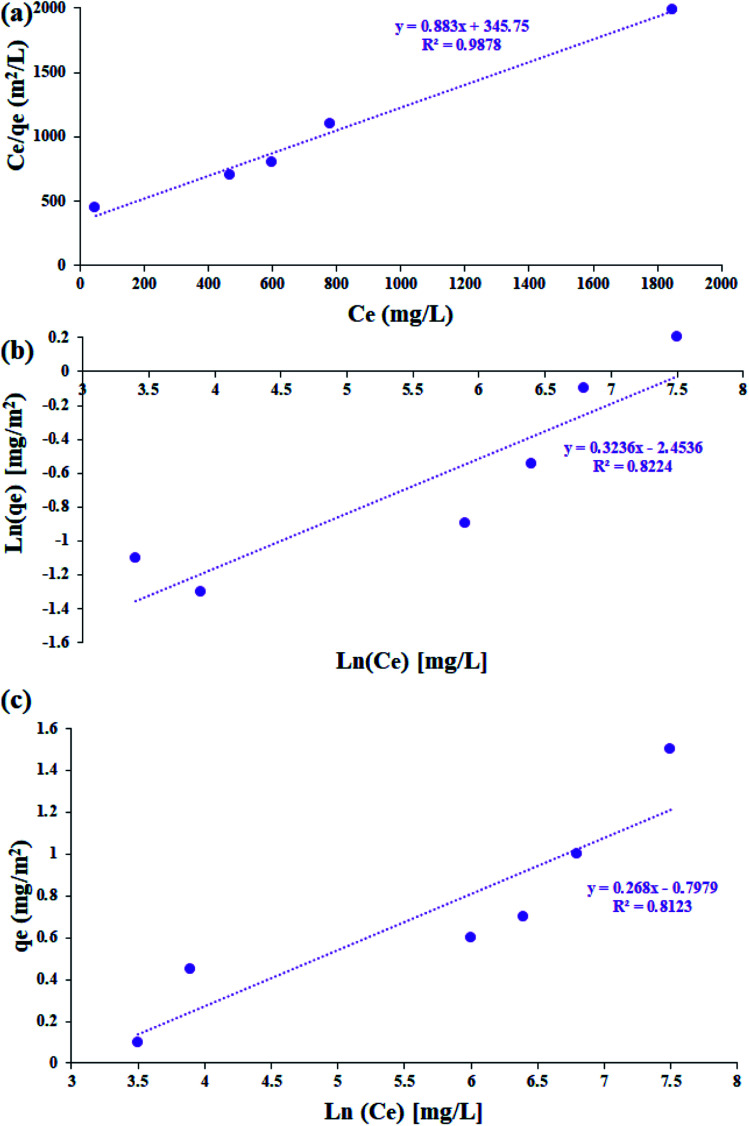
The linearized asphaltene (a) Langmuir, (b) Freundlich, (c) Temkin adsorption isotherms for Fe_3_O_4_@SiO_2_/KCC-1@ZrO_2_/SO_4_^2−^ NPs.

The study of the adsorption equilibrium behavior is necessary for the design and optimization of adsorption processes. In this study, the adsorption isotherm of asphaltene on Fe_3_O_4_@SiO_2_/KCC-1@ZrO_2_/SO_4_^2−^ adsorbent was studied using Langmuir, Freundlich, and Temkin models. Fig. 11 shows the diagrams for each of the isotherms. For this purpose, several experiments with different initial concentrations of asphaltene were performed under optimal conditions for 12 h. The parameters of all three isotherm models along with their correlation coefficients are given in Table 2. The values obtained for 1/*n* in the range of 5 < 1/*n* < 1 indicate that the adsorption is well done; if it is 1 < 1/*n* < 5, adsorption is relatively difficult, and if it is 1/*n* > 1, adsorption is very poor.^33^ As shown in Table 2, the values of 1/*n* for both adsorbents for all three models were less than 1.0, which reflects the desirability of the adsorption process.^33,47^ In addition, by comparing the results of the correlation coefficient (*R*^2^ values), it can be concluded that the Langmuir model has a more suitable range than the Freundlich and Temkin models, which means that the adsorption of asphaltene by both adsorbents occurs through the same mechanism as a single layer by uniform distribution of adsorption sites. This adsorption behavior for Fe_3_O_4_@SiO_2_/KCC-1@ZrO_2_/SO_4_^2−^ is completely consistent with the published reports of asphaltene adsorption behavior on nanoparticles. In the study of asphaltene adsorption on beta-zeolite, Kashefi *et al.* noticed that the Langmuir model describes the adsorption behavior of this process well.^48^ The results showed that the *K*_L_ values for Fe_3_O_4_@SiO_2_/KCC-1@ZrO_2_/SO_4_^2−^ were equal to 0.0040 L mg^−1^, and the maximum asphaltene equilibrium adsorption (*q*_m_) was equal to 1.409 mg m^−2^. Metal oxide NPs, such as magnesium oxide and calcium oxide, also follow the Langmuir isotherm in asphaltene adsorption.^47^ However, Al and Al_2_O_3_ followed the Freundlich isotherm,^28^ which indicates the nature of the NPs in asphaltene adsorption.^28,30,31^

**Table 2:** Estimated parameters for the Langmuir, Freundlich, and Temkin isotherms at 25 °C

Isotherm	Equation	Parameters	
Langmuir	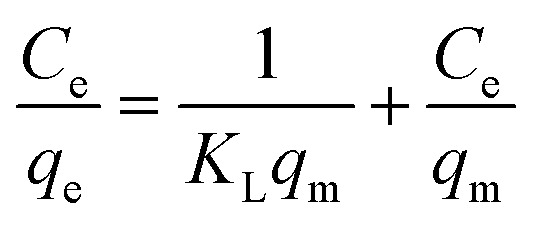	*q*_m_ (mg g^−1^)	1.409
		*K*_L_ (L mg^−1^)	0.0040
		*R* ^2^	0.8123
Freundlich	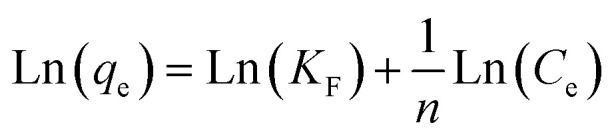	*K*_F_	0.093
		1/*n*	0.444
		*R* ^2^	0.8224
Temkin	*q*_e_ = *B*_1_Ln(*K*_T_) + *B*_1_Ln(*C*_e_)	*K*_T_	0.135
		*B*_1_	0.289
		*R* ^2^	0.8123

According to the measurement of asphaltene adsorption by Fe_3_O_4_@SiO_2_/KCC-1@ZrO_2_/SO_4_^2−^ NPs, their adsorption kinetics can be predicted. To determine the kinetic mechanism of asphaltene adsorption, a duration of 12 h was chosen for this process. At times higher than 12 h, there may be some fluctuations in the adsorption process, although the best time to balance is 24 h.^33,47^ Data fitting with the linear form of the relationships was performed to study the adsorption kinetics of three models: (i) quasi-first-order (eqn (3)), (ii) quasi -second-order (eqn (4)), and (iii) intraparticle diffusion (eqn (5)).^49^ The equations for each are shown below:

Intraparticle diffusion:

3 *q*_*t*_ = *k*_1_*t*^0.5^ + 1

Quasi -second-order:4
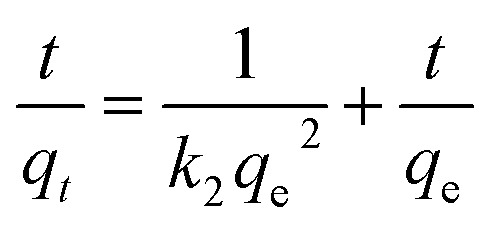


Quasi -first-order:

5 Ln(*q*_e_ − *q*_*t*_) = Ln(*q*_e_) − *k*_1_*t*

Using the slope and *y*-intercept of the curves, the constancy of the kinetic relations of these models was calculated and given in Table 3. The correlation coefficient (*R*^2^) of the data with the kinetic relationships for the quasi-second-order adsorption kinetics for both adsorbents is very close to 1.0. In addition, the experimental values of the equilibrium adsorption of asphaltene *q*_e_ and the values obtained from the quasi-second-order adsorption kinetic relationship are very close to each other (for both adsorbents). As a result, it can be concluded that the asphaltene adsorption by Fe_3_O_4_@SiO_2_/KCC-1@ZrO_2_/SO_4_^2−^ NPs follows the quasi-second adsorption kinetics, and the reaction between the adsorbent surface and the asphaltene is the rate-determining step of the asphaltene adsorption process. Adaptation of the data to the quasi-second-order adsorption kinetics model can be a reason for eliminating the diffusion phase from the adsorption phenomenon, which has led to a reduction in the equilibrium time. The results are consistent with previous studies in this field that have used NPs such as alumina, nickel oxide, and titania, and have followed quasi-second-order adsorption kinetics.^28,30–32,46^

**Table 3:** Kinetic parameters of asphaltene adsorption on Fe_3_O_4_@SiO_2_/KCC-1@ZrO_2_/SO_4_^2−^ NPs

Parameters	*q*_e_ (mg m^−2^)	Kinetic models								
		Quasi-first-order			Quasi-second-order			Intraparticle diffusion		
		*R* ^2^	*k*_1_ (1 h^−1^)	*q*_e_ (mg m^−2^)	*R* ^2^	*k*_2_ (m^2^ mg^−1^ h^−1^)	*q*_e_ (mg m^−2^)	*R* ^2^	*k*_I_ (mg m^−2^ h^−0.5^)	*I*
Value	1.54	0.923	1.138	1.478	0.994	1.439	1.390	0.894	0.728	0.010

### Control tests

In order to clarify the superiority of the Fe_3_O_4_@SiO_2_/KCC-1@ZrO_2_/SO_4_^2−^ adsorbent, the adsorption capability of their raw materials, including Fe_3_O_4_, Fe_3_O_4_@SiO_2_, ZrO_2_/SO_4_^2−^, and KCC-1, under optimal conditions (at room temperature, in optimal amount of 0.7 g L^−1^ and at a concentration of 2000 mg L^−1^ of asphaltene) was studied during the contact time of 12 h.

According to the results, Fe_3_O_4_@SiO_2_, ZrO_2_/SO_4_^2−^, KCC-1, and Fe_3_O_4_@SiO_2_/KCC-1 showed 0.41, 0.62, 0.75, and 1.11 mg m^−2^ asphaltene adsorption, respectively. Fe_3_O_4_ NPs did not show any adsorption during 12 h contact time. By comparing the results with the two adsorbents, it can be concluded that the resulting hybrids with a possible synergistic effect increase the ability of ZrO_2_/SO_4_^2−^ to adsorb asphaltene. The results show that the immobilization of ZrO_2_/SO_4_^2−^ on the Fe_3_O_4_@SiO_2_/KCC-1 framework causes a significant increase in the adsorption capacity of asphaltene molecules.

Although ZrO_2_/SO_4_^2−^ groups have an asphaltene adsorption of 0.62 mg m^−2^, this significant difference with respect to the Fe_3_O_4_@SiO_2_/KCC-1@ZrO_2_/SO_4_^2−^ adsorbent can be directly attributed to the ZrO_2_/SO_4_^2−^ groups immobilized on the fibrous KCC-1. This, along with the spherical structure of the adsorbent nanoparticles, causes a significant increase in surface to volume ratio and consequently a maximum increase in adsorption.

### Reusability

**Fig. 12:**
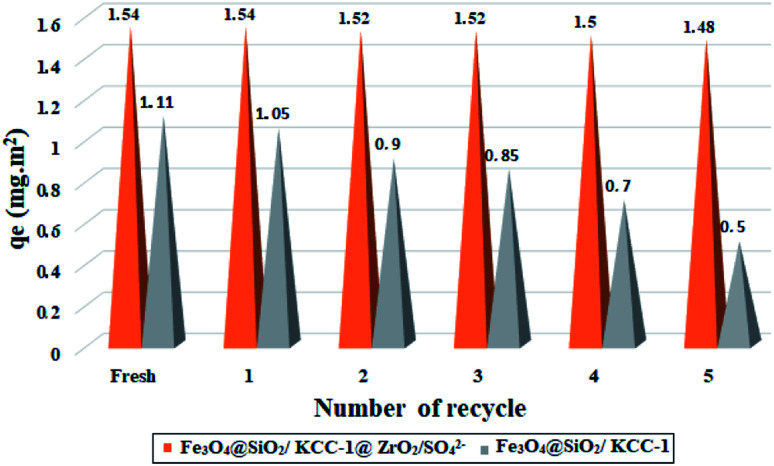
Recyclability of Fe_3_O_4_@SiO_2_/KCC-1@ZrO_2_/SO_4_^2−^ and Fe_3_O_4_@SiO_2_/KCC-1 over the asphaltene adsorption.

In order to show the stability and adsorption activity of the Fe_3_O_4_@SiO_2_/KCC-1@ZrO_2_/SO_4_^2−^ adsorbent, the adsorbent recovery was studied for 5 consecutive cycles. In order to influence the role of the ZrO_2_/SO_4_^2−^ groups, the recoverability of Fe_3_O_4_@SiO_2_/KCC-1 with Fe_3_O_4_@SiO_2_/KCC-1@ZrO_2_/SO_4_^2−^ for asphaltene adsorption was evaluated and compared in successive cycles. The adsorbent was separated from the asphaltene solution at each step by applying an external magnetic field, then washed with water and acid, and reused after heating to 150 °C. Fig. 12 shows the results of the recovery studies under optimal conditions (at room temperature, in an optimal amount of 7 g L^−1^ and at a concentration of 2000 mg L^−1^ of asphaltene) for a contact time of 12 h. A similar value of 0.7 g L^−1^ was used to evaluate the adsorption of Fe_3_O_4_@SiO_2_/KCC-1.

Results were reported based on *q*_e_ (in mg m^−2^). As shown in Fig. 12, Fe_3_O_4_@SiO_2_/KCC-1@ZrO_2_/SO_4_^2−^ has a higher resistance to adsorption properties than Fe_3_O_4_@SiO_2_/KCC-1. Subsequently, negligible activity drops were observed during successive cycles of asphaltene adsorption/desorption. As shown in Fig. 12, the value of *Q*_e_ shifts from 1.54 to 1.48 mg m^−2^ for Fe_3_O_4_@SiO_2_/KCC-1@ZrO_2_/SO_4_^2−^ NPs after 5 consecutive adsorptions/desorption of asphaltene. Meanwhile, this change for Fe_3_O_4_@SiO_2_/KCC-1 increases from 1.11 to 0.5 mg m^−2^ during successive cycles, which is very significant compared to Fe_3_O_4_@SiO_2_/KCC-1@ZrO_2_/SO_4_^2−^.

Due to the acidic and thermal treatment of the adsorbents after each asphaltene adsorption, the results reflect the high stability of the adsorbents while maintaining their adsorption properties. The presence of sulfated zirconium oxide groups not only causes stronger and better adsorption of asphaltene on the adsorbent surface, but the adsorption activity is also maintained during successive cycles. Fe_3_O_4_@KCC-1 NPs appeared to be poisoned during successive cycles, so that even with acid treatment, the catalyst activity was not restored. The high acid stability of sulfated zirconium oxide groups in an acidic environment not only causes high adsorption stability during successive cycles, but also causes the adsorbent to maintain its adsorption activity after acid treatment.

This difference can be directly attributed to the presence of KCC-1 and ZrO_2_/SO_4_^2−^ groups in the Fe_3_O_4_@SiO_2_/KCC-1@ZrO_2_/SO_4_^2−^ adsorbent. Due to its nature, it not only causes stronger and better adsorption of asphaltene on its surface, but the adsorption activity of the adsorbent is also maintained in successive cycles.

In another useful study, BET analysis was performed over the recovered adsorbent before and after acid treatment in successive cycles. As shown in Table 4, the specific surface area of the adsorbent decreased sharply after asphaltene exposure. The remarkable point was the successful recovery of adsorbents after acid treatment in such a way that the specific surface area returns to its original value. This reflects the stability and high activity of the adsorbent, which has maintained its surface properties. According to the results of BET (Table 4), it can be concluded that on average, about 35% of the surface of nanoparticles in each cycle is occupied by asphaltene groups. This amount also reflects the full activation of the surface after each recovery and treatment.

**Table 4:** BET analysis of the fresh and recovered adsorbents before and after treatment

Cycle	Treatment	Adsorbent
		Specific surface area (m^2^ g^−1^)
Fresh	—	120
1st	Before treatment	74
	After treatment	118
2nd	Before treatment	78
	After treatment	122
3rd	Before treatment	78
	After treatment	116
4th	Before treatment	75
	After treatment	118
5th	Before treatment	78
	After treatment	114

**Fig. 13:**
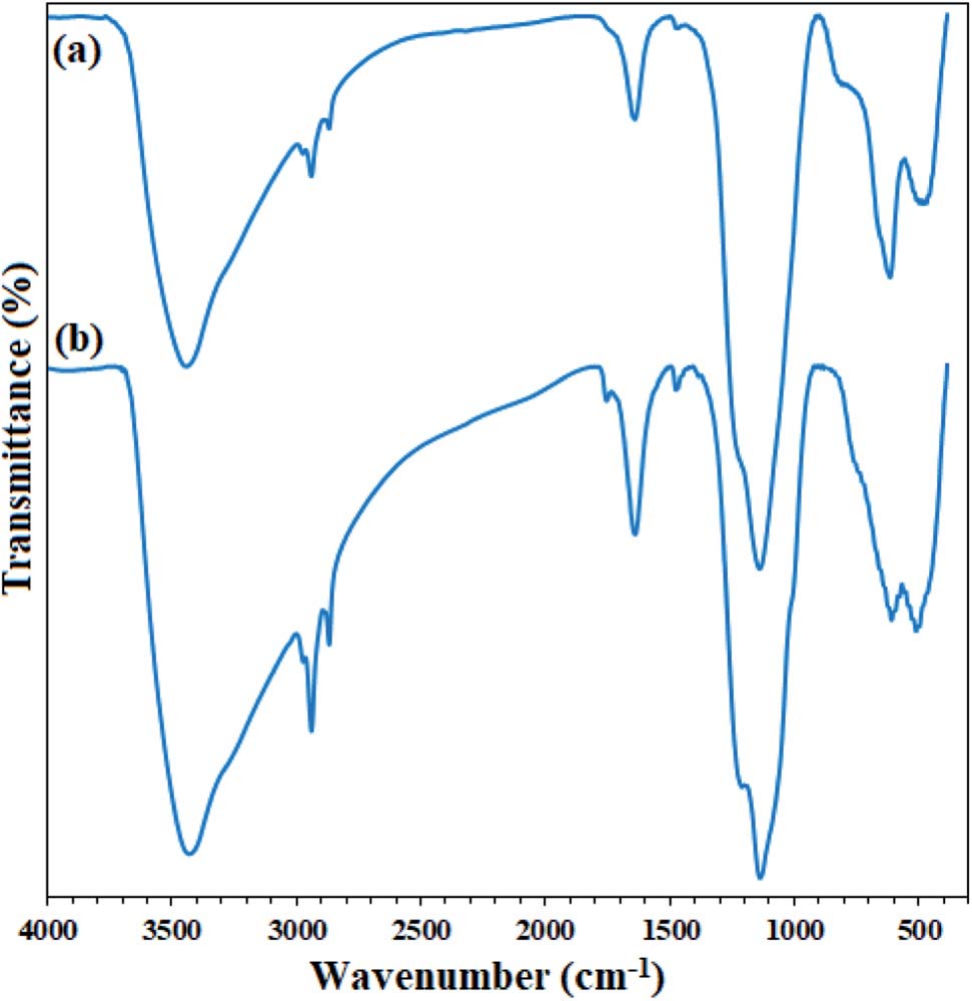
FTIR spectra of (a) freshly prepared Fe_3_O_4_@SiO_2_/KCC-1@ZrO_2_/SO_4_^2−^ and (b) its recovered spectrum after the 5^th^ recycling over the asphaltene adsorption (after treatment).

In addition, FTIR analysis of both recovered adsorbents (after heat and acid treatment) clearly confirms their high stability. As shown in Fig. 13, the FTIR spectra of the adsorbents recovered after the fifth cycle are quite similar to those of the freshly prepared ones. No change in their structure was achieved, despite acidic and thermal treatments.

## Conclusion

In conclusion, a highly efficient, highly durable, and recyclable adsorbent has been developed for the efficient adsorption/removal of asphaltene from crude oil by immobilization of sulfated zirconium oxide (ZrO_2_/SO_4_^2−^) on Fe_3_O_4_@SiO_2_/KCC-1 NPs (Fe_3_O_4_@SiO_2_/KCC-1@ZrO_2_/SO_4_^2−^ NPs). The physical and structural properties of the adsorbent was studied by FTIR, XRD, VSM, BET, FE-SEM, and TEM analyses. The maximum adsorption occurred at ambient temperature in the presence of 0.7 g L^−1^ of the adsorbent at a concentration of 2000 mg L^−1^ of asphaltene. The asphaltene adsorption by the NPs follows a quasi-second order adsorption kinetics, which indicates that the adsorption process is dependent on the asphaltene concentration. Upon isotherm studies over both nanoparticles, the Langmuir model is more efficient than the Freundlich and Temkin models, which means that the adsorption of asphaltene by nanoparticles has a monolayer nature with a uniform distribution of adsorption sites. Another advantage of NPs was their ability to be recovered and reused after acid and heat treatment as an asphaltene adsorbent for several consecutive runs without significant reduction in activity. Due to the acid and heat treatment of both adsorbents after each adsorption of asphaltene, the results reflect the high stability of the adsorbents while maintaining their adsorption properties. Comparative results from control experiments showed that the immobilization of KCC-1 and ZrO_2_/SO_4_^2−^ nanoparticles on the Fe_3_O_4_@SiO_2_ structure significantly increased its adsorption activity towards asphaltene, which was better than Fe_3_O_4_@SiO_2_/KCC-1 and ZrO_2_/SO_4_^2−^. Fe_3_O_4_@SiO_2_/KCC-1@ZrO_2_/SO_4_^2−^ hybrid, with the simultaneous presence of active and adsorbent groups KCC-1 and ZrO_2_/SO_4_^2−^, creates a synergistic effect for the physical adsorption of asphaltene. The results of the control experiments showed that each of the compounds KCC-1 and ZrO_2_/SO_4_^2−^ alone can adsorb asphaltene, but have a significant difference when they are together in the catalyst hybrid. In general, the results show that Fe_3_O_4_@SiO_2_/KCC-1@ZrO_2_/SO_4_^2−^ nanoparticles have the ability to adsorb asphalt under mild conditions, and can be used for this purpose in the relevant industries.

## Conflicts of interest

The authors declare that they have no conflict of interest.

## Supplementary Material
